# Auditory tracts identified with combined fMRI and diffusion tractography

**DOI:** 10.1016/j.neuroimage.2013.09.007

**Published:** 2014-01-01

**Authors:** Faiza Javad, Jason D. Warren, Caroline Micallef, John S. Thornton, Xavier Golay, Tarek Yousry, Laura Mancini

**Affiliations:** a Neuroradiological Academic Unit, Department of Brain Repair and Rehabilitation, UCL Institute of Neurology, University College London, Queen Square, London WC1N 3BG, UK; b Dementia Research Centre, UCL Institute of Neurology, University College London, Queen Square, London WC1N 3BG, UK; c Lysholm Department of Neuroradiology, National Hospital for Neurology and Neurosurgery, UCLH NHS Foundation Trust, Queen Square, London WC1N 3BG, UK

**Keywords:** AC, auditory cortex, AM, amplitude modulation, BOLD, blood oxygenation level dependent, CoG, centre of gravity, cSD, constrained spherical deconvolution, DSI, diffusion spectrum imaging, DTI, diffusion tensor imaging, EPI, echo planar imaging, FDR, false discovery rate, fMRI, functional magnetic resonance imaging, HG, Heschl's gyrus, IndConn, index of connectivity, IC, inferior colliculus, IRN, iterated ripple noise, MGB, medial geniculate body, MNI, Montreal Neurological Institute, MRI, magnetic resonance imaging, PAS, persistent angular structure, PET, positron emission tomography, PP, planum polaris, PT, planum temporalis, SD, spherical deconvolution, SN, substantia nigra, STG, superior temporal gyrus, Auditory tracts, Auditory radiation, fMRI, DTI, Tractography

## Abstract

The auditory tracts in the human brain connect the inferior colliculus (IC) and medial geniculate body (MGB) to various components of the auditory cortex (AC). While in non-human primates and in humans, the auditory system is differentiated in core, belt and parabelt areas, the correspondence between these areas and anatomical landmarks on the human superior temporal gyri is not straightforward, and at present not completely understood. However it is not controversial that there is a hierarchical organization of auditory stimuli processing in the auditory system. The aims of this study were to demonstrate that it is possible to non-invasively and robustly identify auditory projections between the auditory thalamus/brainstem and different functional levels of auditory analysis in the cortex of human subjects in vivo combining functional magnetic resonance imaging (fMRI) with diffusion MRI, and to investigate the possibility of differentiating between different components of the auditory pathways (e.g. projections to areas responsible for *sound*, *pitch* and *melody* processing). We hypothesized that the major limitation in the identification of the auditory pathways is the known problem of crossing fibres and addressed this issue acquiring DTI with b-values higher than commonly used and adopting a multi-fibre ball-and-stick analysis model combined with probabilistic tractography. Fourteen healthy subjects were studied. Auditory areas were localized functionally using an established hierarchical pitch processing fMRI paradigm. Together fMRI and diffusion MRI allowed the successful identification of tracts connecting IC with AC in 64 to 86% of hemispheres and left *sound* areas with homologous areas in the right hemisphere in 86% of hemispheres. The identified tracts corresponded closely with a three-dimensional stereotaxic atlas based on postmortem data. The findings have both neuroscientific and clinical implications for delineation of the human auditory system in vivo.

## Highlights

• We combine fMRI and DTI to successfully identify the auditory pathways in volunteers.
• Tractography results are comparable to postmortem atlas data.
• Clinical fMRI/DTI can successfully identify cortico-subcortical auditory pathways.
• Ascending and descending auditory pathways cannot be differentiated with MRI.

## Introduction

The anatomical and functional organization of the auditory cortical system is still not completely understood, particularly in humans. Current understanding of the auditory processing is based on a variety of experimental approaches (e.g. cytoarchitecture, myeloarchitecture, auditory-evoked potentials, tracer studies, functional magnetic resonance imaging (fMRI) studies and positron emission tomography (PET) studies) in animals (mainly cats and non-human primates) and humans. The auditory system encompasses cortical areas in the superior temporal lobes and subcortical areas including the inferior colliculum (IC) in the brainstem and the middle geniculate body (MGB) in the auditory thalamus. One of the oldest and best characterised organizational features in the auditory system is its cochleotopic or tonotopic organization, which refers to the ordered representation of sound frequency in auditory areas. Tonotopy has been shown at all levels of the auditory pathway, including the cochlea, the auditory brainstem nuclei and the auditory cortex in mammals (including non-human primates and humans) and birds. Despite there being an agreement with the presence of tonotopic organization, over the years tonotopy studies have reached different conclusions as to the precise localization of the boundaries between areas specific for low and high frequency tones. In a recent review Baumann et al. (2013) proposed a robust scheme of interpretation of all previous tonotopic studies that reconciles the apparently conflicting evidence of past tonotopic studies and emphasizes the similarities of the human tonotopic maps to those in non-human primates. It is not controversial, on the basis of anatomical and physiological characteristics, that the auditory cortex in non-human primates and in humans includes a tonotopic core region which represents a first stage of processing, surrounded by a belt of tissue that is less clearly tonotopic, and flanked further by auditory areas that show no evidence of tonotopy (Baumann et al., 2013, Brodal, 1981, Hackett et al., 2001, Semple and Scott, 2003). Core, belt and parabelt regions have also been described as having distinctive histological compositions, specific thalamocortical and corticocortical connections, and unique physiological and functional characteristics (Baumann et al., 2013, Fullerton and Pandya, 2007, Galaburda and Pandya, 1983, Galaburda and Sanides, 1980, Morel et al., 1993, Morosan et al., 2001, Morosan et al., 2005, Wallace et al., 2002). In humans a different nomenclature has been adopted, with a ‘primary auditory cortex’ (PAC) identified in portions of the transverse temporal Heschl's gyrus (HG), located on the supratemporal plane of the superior temporal gyrus (STG), and other auditory areas identified in the planum polaris (PP) on the STG anterior to HG and in the planum temporalis (PT) on the STG posterior to the HG (Fullerton and Pandya, 2007). However, the correspondence between these areas (PAC, HG, PP, PT) and the core, belt and parabelt areas is not straightforward (Baumann et al., 2013).

It has also been shown that increased stimulus complexity is associated with increased activation throughout the auditory cortical core and surrounding auditory areas (Patterson et al., 2002, Semple and Scott, 2003), and it is not controversial that the auditory system presents a neural hierarchy of melody processing in the auditory pathway (Griffiths et al., 1998, Howard et al., 2000, Patterson et al., 2002). Positron emission tomography (PET) and fMRI studies have revealed activation of HG for example when listening to any type of sound compared to silence, while sounds with complex spectro-temporal structure (including modulation of frequency or amplitude) activate areas immediately anterolateral to the core, in what is probably belt or parabelt (Patterson et al., 2002, Scott and Johnsrude, 2003). Lateral to HG, activation has been observed in response not only to harmonic tones and sounds with changing spectral structure but also to phonetic cues and intelligible speech. Currently, speech-related activations in regions lateral to HG cannot be spatially distinguished from responses to non-speech sounds with spectral details of variation (Patterson et al., 2002, Scott and Johnsrude, 2003).

Subcortically, ascending and descending projections connect auditory receptors with cerebral cortex via various auditory centres: the cochlear nuclei, the superior olive, the inferior colliculus (IC) and the medial geniculate body (MGB). These relays in the propagation of auditory information also mediate its interaction with other information (e.g. other sensory modalities) (Brodal, 1981, Moller, 2006, Palmer and Rees, 2010).

Between IC and cortex, the ascending projection connects portions of the IC with portions of the MGB and then with the auditory cortex. The descending projections are anatomically parallel to the ascending projections, but the corticocollicular projection bypasses the MGB on its way to the IC (Andersen et al., 1980, Brodal, 1981, Calford and Aitkin, 1983, Lee and Sherman, 2010, Llano and Sherman, 2008, Moller, 2006, Palmer and Rees, 2010). Commissural connections are present at the IC and corpus callosum levels (Brodal, 1981, Code and Winer, 1985).

Identification of the human auditory tracts is of clinical (e.g. pre-operative assessment before cochlear implantation or brain surgery) as well as neurobiological importance. However, to date only a single study has used functional and anatomical information (Crippa et al., 2010) to identify auditory tracts with low reproducibility (in 50% of subjects) under the hypothesis that the main limitation to their identification is image resolution.

Diffusion MRI is the only currently available method enabling non-invasive identification of white matter structures in vivo, by measuring the diffusion of water molecules in the brain (Basser et al., 1994, Pierpaoli et al., 1996). Since axon radii are in the range of 0.1–10 μm, whereas typical voxel has sides in the range of 1–5 mm, voxels contain hundreds of thousands of axon fibres, which can adopt a wide range of often complex configurations. Diffusion MRI presents known limitations in resolving multiple fibre populations in each image voxel (also known as crossing and kissing fibres problem; Behrens et al., 2003, Seurinane and Alexander, 2009), which has so far limited its application in the identification of the auditory white matter fascicles connecting the IC and MGB to the auditory cortices. In fact, although white matter pathways cross all over the brain, the auditory radiation is particularly susceptible to this problem due to its small size and to its crossing, along its path, with larger fascicles such as the cortico-spinal tracts and the optic radiation. Several methods have been implemented to surmount the problem of resolving multiple fibres, including two-tensor and ball-and-stick models, persistent angular structure (PAS) MRI, spherical deconvolution (SD), constrained spherical deconvolution (cSD), diffusion spectrum imaging (DSI) and Q-ball imaging (Seurinane and Alexander, 2009). All these methods have different acquisition and computation time requirements, different accuracies and levels of bias. For example DSI and PAS–MRI have the highest accuracy, but DSI acquisitions are very demanding (e.g. 515 diffusion encoding steps, 48 min acquisition time on a 3 T scanner), while PAS–MRI is computationally very demanding (Sakaie, 2008, Seurinane and Alexander, 2009).

The main aims of the present study were to identify the auditory radiation connecting IC and MGB with the auditory cortex using a combined fMRI/DTI approach in a clinical scanner, with acquisition parameters tolerable in patients, and to assess (i) the robustness of the identification, (ii) the possibility of differentiating between components of the acoustic radiation, and (iii) comparison of the results with available 3D stereotaxic atlas. A secondary aim was to investigate if also inter-hemispheric connections could be visualised with the same methodology.

fMRI was used to help differentiating auditory functional cortical zones that are likely a priori to have different profiles of subcortical projections, to overcome the difficulties in the localization of these cortical zones from anatomical landmarks alone. The selected fMRI paradigm is a sensitive protocol known to identify a hierarchical representation in the auditory cortex (Patterson et al., 2002). It is based on the assumption that, from the auditory perspective, perception of a melody (a sequence of notes like that produced when someone picks out a tune on the piano with one finger) involves: (1) detecting temporal regularities in segments of an extended sound, (2) determining the pitch of each of these regular segments, and (3) determining how the pitch changes from note to note over the course of the sound. It includes four different stimuli (silence, noise without pitch, iterated ripple noise (IRN) with fixed pitch and IRN with varying pitch). Their combinations activate areas in the central portion of HG, largely symmetrical in both hemispheres, when all three sound conditions are compared to the silence baseline, areas in the antero-lateral HG (symmetrical in both hemispheres) when all conditions with pitch are compared to the pitchless noise condition, and areas in STG and PP (asymmetric, with more activation on the right hemisphere) when the condition with varying pitch is compared with the condition with fixed pitch (Patterson et al., 2002).

For the diffusion MRI, a multi-tensor ball-and-stick model was selected, in combination with probabilistic tractography, which showed promising results in previous studies (Behrens et al., 2007, Crippa et al., 2010). This method is characterised by low acquisition requirements (64 gradient diffusion directions and a b-value of 1400 s/mm^2^), medium computation times, medium accuracy and low bias (Seurinane and Alexander, 2009), and it can therefore be implemented on a clinical scanner for routine MRI acquisitions in patients.

To assess whether the obtained fascicles were plausible, results were directly compared with a stereotaxic 3D representation of the human auditory radiation computed from myeloarchitectonic and cytoarchitectonic maps (Rademacher et al., 2002).

## Materials and methods

### Subjects

Fourteen healthy right-handed volunteers (8 female, mean (standard deviation) age 24.7 (2.4) years) with no history of neurological or hearing disorders participated in the study, which was approved by the National Hospital for Neurology and Neurosurgery & Institute of Neurology Joint Research Ethics Committee. All subjects gave informed written consent prior to their participation.

### Data acquisition

Images were acquired with a Siemens 3T TIM Trio MRI system (Siemens, Erlangen, Germany). Each scan session lasted 100 min.

#### Structural imaging

Sagittal 3-dimensional T1-weighted magnetization-prepared rapid gradient-echo (MPRAGE) sequences of the whole brain were acquired with one slab of 176 slices with slice thickness = 1.1 mm; gap = 0.55 mm; in-plane resolution = 1.1 × 1.1 mm^2^, inversion time TI = 900 ms, flip angle = 10°, and repetition time/echo time (TR/TE) = 2200/2.88 ms. These images were required for the identification of subcortical regions of interest to seed tractography, and to create a group-average T1-weighted image in standard space on which to overlay functional data.

#### fMRI

Functional brain images were acquired using a blood oxygenation level-dependent (BOLD) sensitive sequence: gradient echo - echo planar imaging (GE-EPI), TR/TE = 11000/30 ms, tilted = 30° from AC–PC line, FOV = 192 × 192, matrix = 96 × 96, in-plane resolution = 2 × 2 mm^2^, slice thickness = 2 mm, gap = 1 mm, with 48 slices covering the whole brain, bandwidth = 2265Hz/Px. A sparse MR acquisition protocol was used (Hall et al., 1999); auditory stimuli were presented during the inter-scan interval (duration 8 s).

#### Field maps

For distortion correction magnetic field maps were acquired with 64 slices (tilted = 30° from the AC–PC line), slice thickness/gap = 3 mm/0 mm, TR = 688 ms, TE1/TE2 = 4.92/7.38 ms, FOV = 240 × 240, and matrix = 80 × 80 with resolution of 3 × 3 × 3 mm^3^.

#### DTI

Two diffusion tensor imaging (DTI) datasets were collected using a diffusion-weighted spin-echo double refocused EPI sequence, 64 diffusion-sensitising gradient directions with b = 1400 s/mm^2^, 64 slices tilted by 30° from the AC–PC line, slice thickness/gap = 2.3 mm/0 mm, TR/TE = 7900/90 ms, FOV = 220 × 220, matrix = 96 × 96, resolution of 2.3 × 2.3 × 2.3 mm^3^, parallel imaging (GRAPPA with acceleration factor of 2), and bandwidth = 1578Hz/Px with acquisition time of 8 min 59 s for each dataset. A total of 10 images with b = 0 s/mm^2^ were also acquired.

### fMRI paradigm

Auditory stimuli were presented via an Esys fMRI system (Invivo Corporation, Orlando, FL, USA) equipped with a 30′′ MR safe, RF shielded, LCD display visible to the subject through a mirror while lying supine within the scanner and with pneumatic headphones embedded in ear-defenders (~ 25 dB passive noise attenuation). The system is driven by a magnetically shielded stimulus delivery computer running E-Prime® software (Psychology Software Tools, Inc, Pittsburgh, PA, USA).

#### Auditory stimuli

The auditory stimuli presented in the fMRI paradigm were adapted from Patterson et al. (2002) in which pitch properties of sound sequences were manipulated to produce hierarchical activation of the central auditory system. Auditory stimuli were Western diatonic music and synthetic sounds created digitally as wave files under Matlab 7.0® (The Mathworks, Massachusetts, USA). Synthetic sounds were based on fixed amplitude random phase noise (bandwidth 0–22 kHz). A subset of these stimuli had temporal pitch created from iterated ripple noise (IRN), whereby copies of a random noise were added iteratively to the original noise waveform with a fixed delay; the resulting composite noise waveform has a pitch equivalent to the reciprocal of the delay latency (Patterson et al., 2002). IRN stimuli with pitch 100, 120, 140, 160, 180, 200, 220, 240, 260 and 280 Hz were created. All synthetic sounds had amplitude modulation (AM) applied at 30 Hz (modulation depth 40%). IRN and AM stimuli were used as these were anticipated to be effective in activating subcortical auditory structures including the MGB (Giraud et al., 2000, Griffiths et al., 2001). Synthetic noise and IRN sounds, each 1 s in duration, were concatenated with an inter-sound gap of 0.1 s into stimulus sequences containing 7 sounds. Average (root-mean-square) intensity was fixed for all stimuli. Three types of sound sequence were synthesised, corresponding to three stimulus conditions: noise sequences with no pitch (noise), IRN sequences with fixed pitch (IRN fixed pitch), and IRN sequences with randomly varying pitch (IRN varying pitch).

#### fMRI paradigm

Four experimental conditions were presented, interleaved in a mini-block design: noise (condition 1); IRN fixed pitch (condition 2); IRN varying pitch (condition 3); and silence (rest, condition 4).

**Fig. 1:**
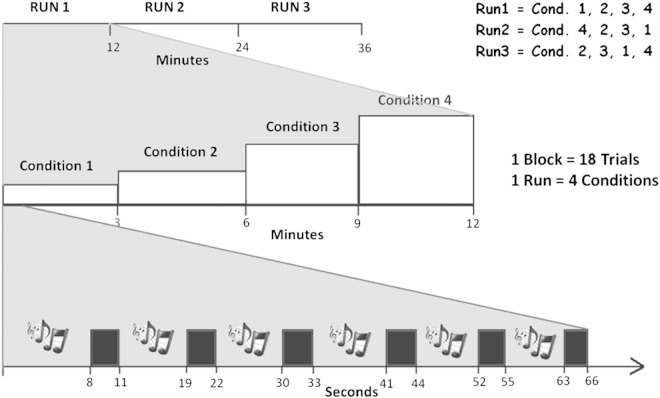
Diagrammatic representation of the experimental fMRI paradigm. Three runs were acquired, each with 4 conditions: 3 sound conditions (noise, fixed-pitch, varying-pitch) and 1 silent or baseline condition. Conditions were presented randomly in each run. The order of the conditions' presentation in each run was: 1 2 3 4 in run 1; 4 2 3 1 in run 2; and 2 3 1 4 in run 3. Condition 1 = noise, Condition 2 = fixed-pitch, Condition 3 = varying pitch and Condition 4 = silence. A sparse acquisition was used in which a stimulus is presented for 8 s while the MRI scanner is paused, followed by 3 s of data acquisition. The timings in the diagram are approximated; precise timings are reported in the Methods section.

Three separate fMRI runs were acquired, the acquisition time of each run being approximately 14 min. Fig. 1 shows a diagrammatic representation of the paradigm. In each run 4 blocks, each of duration 3 min 18 s, with stimulation comprising noise, IRN fixed pitch, IRN varying pitch, and silence were acquired, for a total acquisition time of 13 min 47 s. The order of the 4 conditions was different in each run: conditions 1 2 3 4 in run 1; 4 2 3 1 in run 2; and 2 3 1 4 in run 3. In summary, a total of 216 functional image volumes were acquired with each condition comprising 54 trials (Fig. 1). During the measurements the scanner room lights were dimmed and the subject was presented with a fixation cross. Subjects were asked to pay attention to the sounds while fixing visually on the cross.

### Data analysis

Data analysis was performed on both individual and group bases. Intra-individual analysis becomes important as some auditory structures are small and show wide inter-individual variation (Campain and Minckler, 1976, Menendez-Colino et al., 2007). The fMRI activations enabled us to locate auditory processing cortical areas with which to seed fibre tractography. Regions of interest in the subcortical areas of the IC and MGB were identified anatomically, as described further down. The fibre tractography was performed in two well-defined steps; the first was to estimate the diffusion tensor of each voxel and the second to define the starting region (seed ROI), termination and waypoint regions to perform the tract determination.

#### Group T1 image

Structural data were processed and analyzed using SPM8 (www.fil.ion.ucl.ac.uk/spm). T1 images for each subject were spatially normalised to Montreal Neurological Institute (MNI) stereotactic space using the SPM8 segmentation toolbox. The spatially normalised images were then averaged to create a group T1 image specific to our subject group.

#### fMRI analysis

**Fig. 2:**
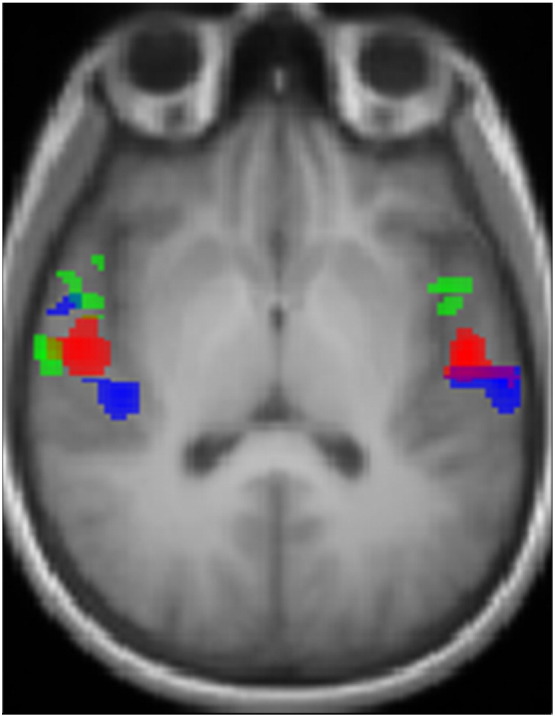
Group fMRI activation overlaid onto the group-average T1 image in MNI template space. The slice is oriented parallel to the planum temporalis and is in radiological convention. Blue = *sound* activation corrected for multiple comparison with FDR p < 0.004; red = *pitch* activation at uncorrected p < 0.001; green = *melody* activation at uncorrected p < 0.001.

Functional data were also processed and analyzed using SPM8. The BOLD time series was realigned to the first image of the series and unwarped using fieldmap data to correct for distortions. The realigned BOLD images were coregistered to the T1 image and spatially normalised to the MNI stereotactic space using the transformation matrix obtained for the spatial normalisation of the T1 image (as described in the previous section). Normalised EPI images were smoothed with an isotropic Gaussian kernel of 4 mm full-width at half maximum. The evoked hemodynamic response for each stimulus was modelled as a boxcar convolved with a synthetic hemodynamic response function in the context of the general linear model. Contrasts between conditions of interest were estimated using a random effects model and enabled the identification of areas activated by ‘*sound*’, ‘*pitch*’, and ‘*melody*’. *Sound* areas (brain regions with sensitivity to sound) were identified by the contrast of all sound minus silence; *pitch* areas (regions with increased sensitivity to pitch information) by the contrast of all sounds with pitch minus noise and *melody* areas (regions with increased sensitivity to pitch pattern in melodies) by sounds with varying pitch minus fixed pitch. *Sound* areas activation map was obtained with correction for multiple comparison false discovery rate (FDR) with p value 0.004. *Pitch* and *melody* areas activation maps were obtained with uncorrected p value 0.001 (Fig. 2). Subcortical activation was visualised in the all sounds minus silence contrast with uncorrected p value 0.01.

#### Tractography seed ROIs

##### Cortical ROIs

**Fig. 3:**
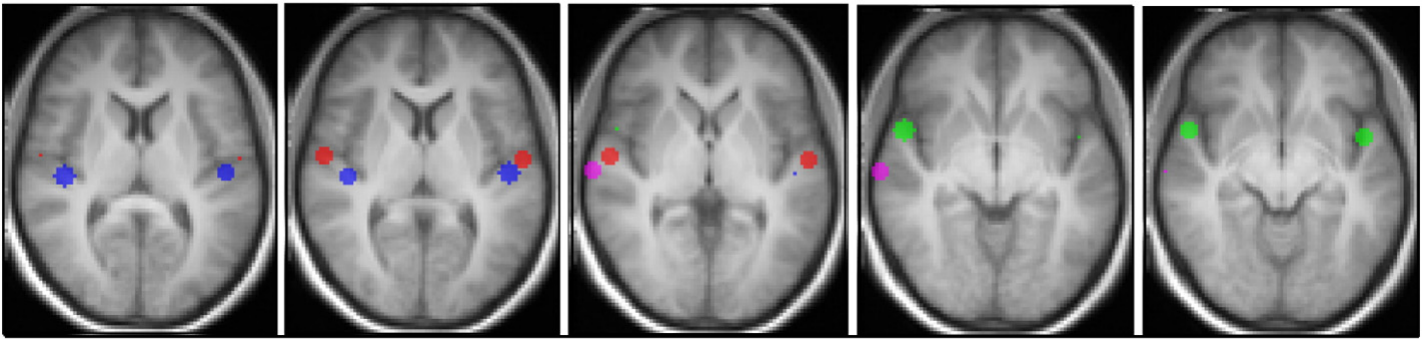
Cortical regions of interest (ROI) used for tractography, overlaid onto the group-average T1 image of our subject, in MNI space. Images are show in radiological convention. The ROIs are spheres of 6 mm radius centred in the CoG of the fMRI activation maps. Blue = *sound* areas, red = *pitch* areas, green = anterior *melody* area, pink = posterior *melody* areas.

Cortical activation maps were thresholded at t-values of 3, 5 and 7. The centre of gravity (CoG) for each activation area was then calculated for each threshold with the fslstats toolbox of the FSL software (www.fmrib.ox.ac.uk/fsl/). Functional activation was localized based on the geometric centre of gravity of the activation map (Alkadhi et al., 2002). For each activation map, the coordinates of the CoG were calculated for the three selected t-thresholds and the average of the three measurements was selected as CoG location. Spheres of radius 6 mm centred on the CoG in each case were created for the primary, secondary and association cortices in MNI space (Fig. 3) and then transformed to single-subject T1-weighted image space with the reverse transformation obtained with the SPM segmentation toolbox. This radius dimension was selected arbitrarily to yield a sphere that could be contained within Heschl's gyrus taking into account that spatial agreement between activation on fMRI and direct intra-operative cortical stimulation is of this order (Bizzi et al., 2008, Yetkin et al., 1997, Yousry et al., 1995).

##### Subcortical ROIs

Four subcortical ROIs (left-IC, left-MGB, right-IC and right-MGB) were defined with the ITK-SNAP software (www.itksnap.org) using anatomical landmarks in the native space of each participant's high-resolution T1-weighted MRI images.

**Fig. 4:**
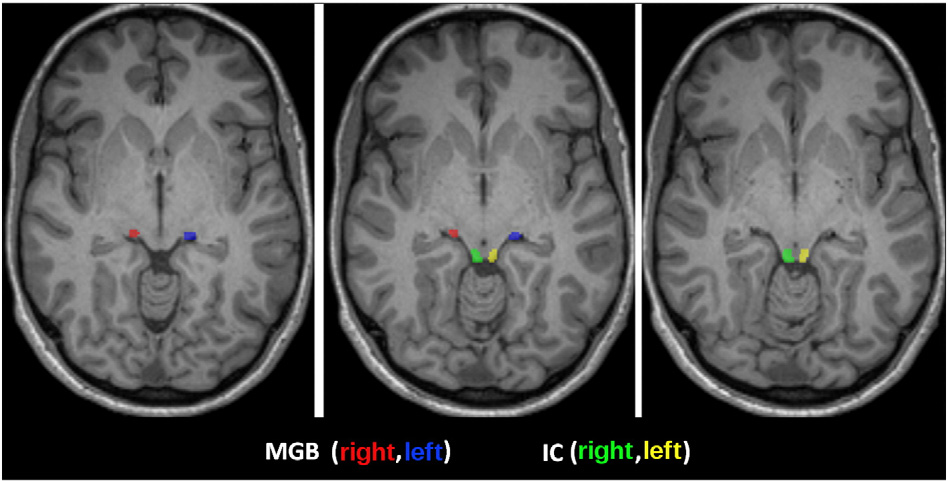
Example of the selected IC and MGB sub-cortical areas on the T1 image of a volunteer. Images are in radiological convention. Green = right IC, yellow = left IC, red = right MGB, blue = left MGB.

The ICs were readily identified as distinct anatomical circular areas on axial views, and as focal elevations on coronal and sagittal planes. Each MGB was identified independently using the following procedure: first the coronal slice showing the substantia nigra (SN) meeting at the interpeduncular fossa was selected. The SN appears as a region of high intensity running infero-medially from the thalamus. The lateral geniculate body was identified on this slice, and the MGB appeared immediately medial to the lateral geniculate body as an oval region of low intensity. The dorsomedial border of the MGB was less clear but was completed manually using the assumption that the shape of the MGB is roughly ovoid (Hirai and Jones, 1989). MGB was also identified as being a small focal elevation projecting from the dorsal thalamus at the level of junction between the inferior and the superior collicular region on the sagittal plane. On average, ROIs comprised 10 and 7 ROI voxels for the IC and MGB respectively. Examples of the IC and MGB ROIs are shown in Fig. 4.

For each subject, after identification of cortical and subcortical ROIs, all regions were transformed from the native T1 space to the native DTI space to be used as seed and control ROIs for the tractography. The transformation was performed using the FLIRT toolbox in the FSL software, with a rigid body 6 parameters model and normalised mutual information as cost function.

#### DTI analysis

Single subject DTI and fibre tracking analyses were performed using FSL software (www.fmrib.ox.ac.uk/fsl). The steps undertaken for DTI data analysis included: 1) Correction of eddy current distortion, 2) Creation of brain mask with skull exclusion, 3) Local fitting of a 2 diffusion tensor model for each voxel, and 4) Probabilistic tractography with 5000 streamlines started at each seed ROI voxel. Tractography was performed from seed to target ROIs as well as from target to seed ROIs to reduce the technical known limitation of the tracking process by which the confidence assigned to connections diminishes with distance from the starting point (Jones, 2011). Tractography was performed separately between the *sound*, *pitch* and *melody* cortical areas (seed ROIs) and the IC (target ROI) both directly or via the MGB (used as waypoint) in both hemispheres. The reverse tractography was also calculated, separately connecting the IC (seed ROI) with the *sound*, *pitch* and *melody* cortical areas (target RoIs) both directly and via the MGB (waypoint) in both hemispheres. Tractography was also performed to identify tracts connecting *sound* areas in the left hemisphere with homologous areas in the right hemisphere and vice versa. Each tractography result was then normalised to the number of streamlines reaching the target ROIs such that the signal intensity in each voxel represents the index of connectivity (IndConn) of that voxel with the seed and target areas. Fibres connecting the same seed and target ROIs in reverse directions (e.g. IC as seed ROI to *sound* area as target ROI and *sound* area as seed ROI to IC as target ROI etc.) were then averaged to account for any discrepancies inherent to the process of probabilistic tractography. Six final tracts were obtained for each hemisphere separately connecting *sound*, *pitch* and *melody* areas with IC either via MGB (ascending auditory projections) or directly (descending projections), and one tract connecting L-*sound* area and R-*sound* area. The tracts were then transformed from native DTI to native T1 space with FLIRT and then from native T1 space to MNI space with SPM8. IndConn thresholds of 1% and 5% were then applied separately to each tract. The tracts thresholded at 5% were then binarized, and group tracts were obtained by co-adding corresponding thresholded and binarized tracts from different subjects.

#### Comparison of tractography results with 3D atlas

The group tracts identified with the tractography method outlined above were compared to a three-dimensional stereotaxic atlas based on microscopically defined localization and topographic data from myelin-stained histological sections of ten postmortem brains (Burgel et al., 2006). For a qualitative visual comparison, the atlas tracts were overlaid onto the group T1 image specific to our subject group, together with the group tracts identified with our processing method. Quantitative measures of inter-subject variation were obtained by calculating the mean and standard deviation of the group tracts and of the atlas tracts in the imaging space of the group T1.

## Results

### fMRI results

To investigate activation patterns due to the different stimuli in each subject, we constructed 3 contrasts at group level. These contrasts gave peak activations in the *sound*, *pitch* and *melody* areas, with coordinates detailed in Table 1.

**Table 1:** Coordinates of peaks in the fMRI activation maps with all three contrasts.

fMRI contrast	Anatomical area	Left			Right		
		x	y	z	x	y	z
All sound minus silence	Postero-medial HG	− 52	− 22	4	44	− 22	10
	Antero-lateral HG	− 64	− 18	10	60	− 2	− 5
All sounds with pitch minus noise	Antero-lateral HG	− 54	− 12	4	56	− 10	4
	Cerebellum				26	− 82	50
All sounds with varying pitch minus fixed pitch	Anterior STG	− 46	2	11	58	6	− 2
	Posterior STG				66	− 16	1

HG = Heschl's gyrus, STG = superior temporal gyrus.

*Sound* areas were identified by the contrast of all three sound conditions over the silence baseline. This revealed extensive activation in bihemispheric cortical areas including postero-medial HG and surrounding superior temporal lobe regions. Peak activations for this contrast are shown as blue areas in Fig. 2, in which the fMRI auditory activation is overlaid on the group-average T1 image. The activation in the postero-medial Heschl's gyrus was used to localize the *sound* area, and the centre of gravity of this activation region used to define the *sound* spherical seed region for tractography (blue area in Fig. 3).

*Pitch* areas were localized by the contrast of all conditions with pitch (fixed IRN pitch and varying IRN pitch) over the pitchless noise condition. It revealed bihemispheric activation in antero-lateral HG (red areas in Fig. 2). This activation was used to localize the *pitch* areas (corresponding to the cortical ‘pitch centre’ identified by Patterson et al. (2002)) and create the *pitch* spherical seed region for tractography (red area in Fig. 3). The inverse contrast of pitchless noise over pitch conditions produced no activation.

*Melody* areas were identified by the contrast of conditions with variable pitch over the fixed IRN pitch condition. It revealed activation in STG (green areas in Fig. 2). The anterior STG was activated bilaterally while the STG area posterior to the HG was activated only on the right hemisphere. All three areas on the STG were used to localize *melody* areas, measure its centre of gravity and create the *melody* spherical seed region for tractography (green and pink area in Fig. 3). The inverse contrast of fixed-pitch over melody produced no activation.

Statistical parametric maps for each of the contrasts of interest were assessed in each individual subject in order to assess the extent of inter-individual variation in activation and functional area localization in relation to the subject group as a whole. The profile and distribution of activation for each contrast in individual subjects were in general similar to the group as a whole. This suggests that it is valid to use localization data derived from the group analysis to make anatomical inferences at single subject level for DTI analysis.

### DTI results

#### General results

Seven separate auditory fascicles were successfully identified in 20 to 24 out of 28 hemispheres (71% to 86% of hemispheres) using an IndConn threshold of 1% and in 18 to 24 out of 28 hemispheres (64% to 86% of hemispheres) using an IndConn threshold of 5%, as detailed in Table 2. Fascicles connecting the *sound*, *pitch* and *melody* cortical areas directly with the IC were successfully identified in a lower number of hemispheres (20–21 out of 28 hemispheres (71–75%) with IndConn threshold of 1%, 18–19 out of 28 hemispheres (64–68%) with IndConn threshold of 5%) than pathways passing through the MGB (22–24 out of 28 hemispheres (79–86%) with IndConn threshold of either 1% or 5%). Tracts connecting left and right *sound* areas were identified in 24 out of 28 hemispheres (86% of hemispheres).

**Table 2:** Number and percentage of hemispheres with successful tractography at two different index of connectivity (IndConn) thresholds (thr).

	Tracts directly to IC (Nr of hemispheres out of 28, %)			Tracts to IC via MGB (Nr of hemispheres out of 28, %)			Inter-hemispheric
	Sound	Pitch	Melody	Sound	Pitch	Melody	L *sound* — R *sound*
IndConn thr 1%	21 (75%)	20 (71%)	20 (71%)	24 (86%)	24 (86%)	22 (79%)	24 (86%)
IndConn thr 5%	18 (64%)	18 (64%)	19 (68%)	24 (86%)	23 (82%)	22 (79%)	24 (86%)

Since the group tracts were obtained by co-adding corresponding thresholded and binarized tracts from different subjects, the signal intensity in the group fascicles is proportional to the number of subjects in which the fascicles were identified. Group fascicles are therefore also a representation of inter-subject variability.

The tracts from the IC level move in a curvilinear fashion upwards towards the auditory cortex (AC) and connect to it as shown in Fig. 6, Fig. 7, Fig. 8, Fig. 9, Fig. 10, where all fascicles are superimposed on the 14 subjects' template T1-weighted group image. In Fig. 6, Fig. 7, Fig. 8, Fig. 9 the slices from left to right move upward from IC to the auditory cortex levels.

**Fig. 5:**
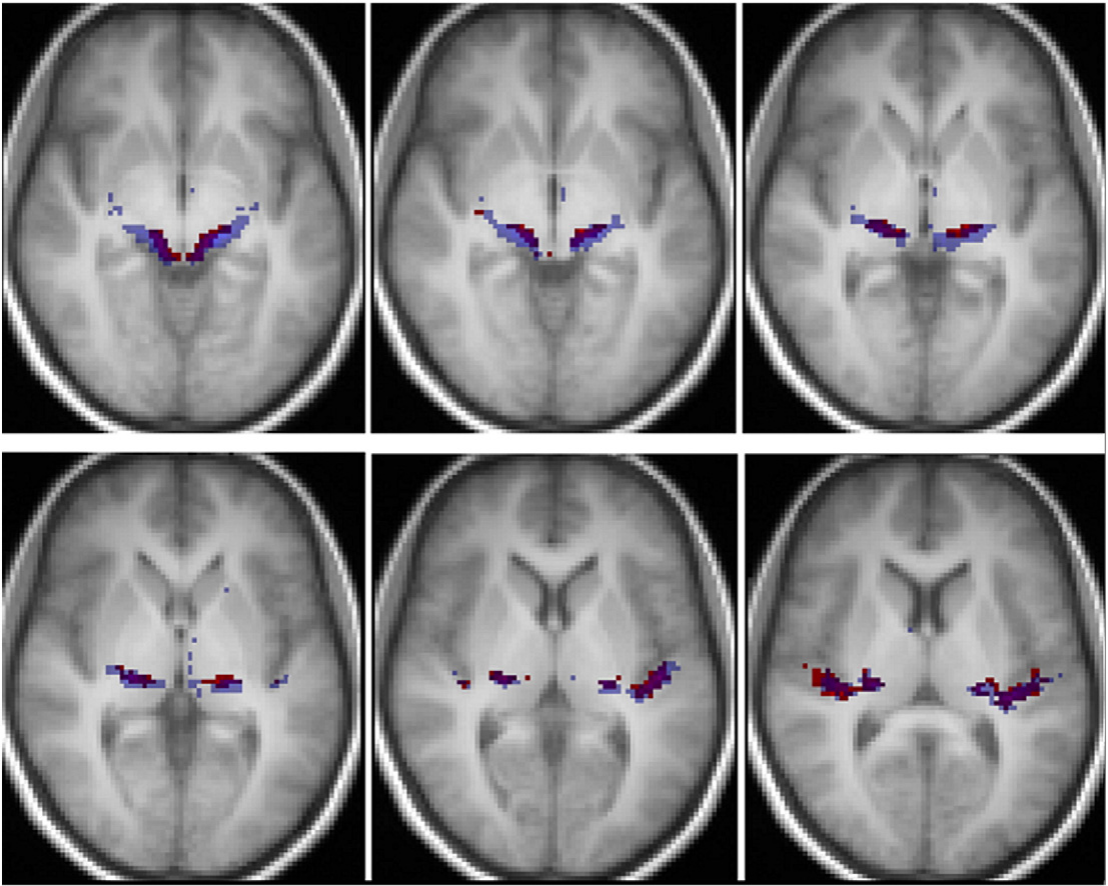
Group tracts connecting IC to the *sound* area directly (red tracts) and via the MGB (blue tracts), overlaid onto the group-average T1 image. Images are in radiological convention.

**Fig. 6:**
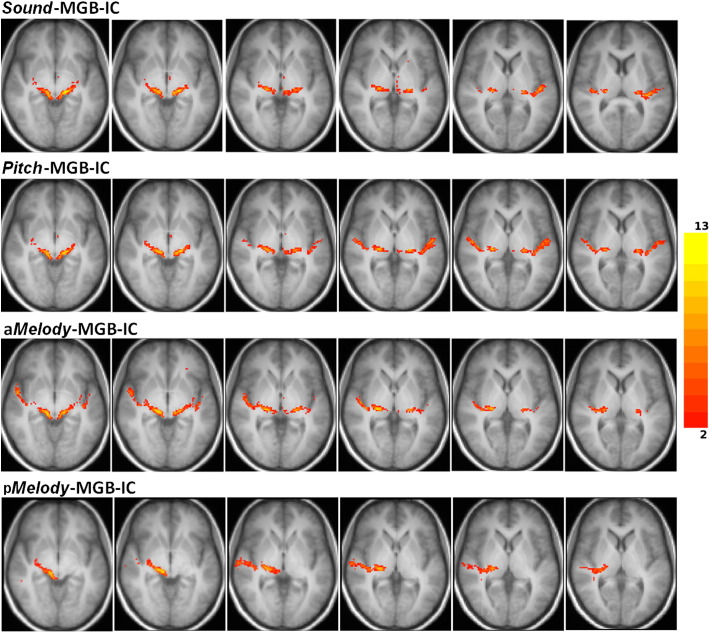
Group fascicles for connections of *sound*, *pitch* and *melody* areas of the auditory cortex to IC via MGB, overlaid onto the group-average T1 image. Images are in radiological convention. The signal intensity is proportional to the number of subjects in which the tracts were identified. Only tracts identified in two or more subjects are depicted. Tracts connecting: *sound*–MGB–IC in the first row, *pitch*–MGB–IC in the second row, anterior *melody* area–MGB–IC in the third row, posterior *melody* area–MGB–IC in the fourth row.

**Fig. 7:**
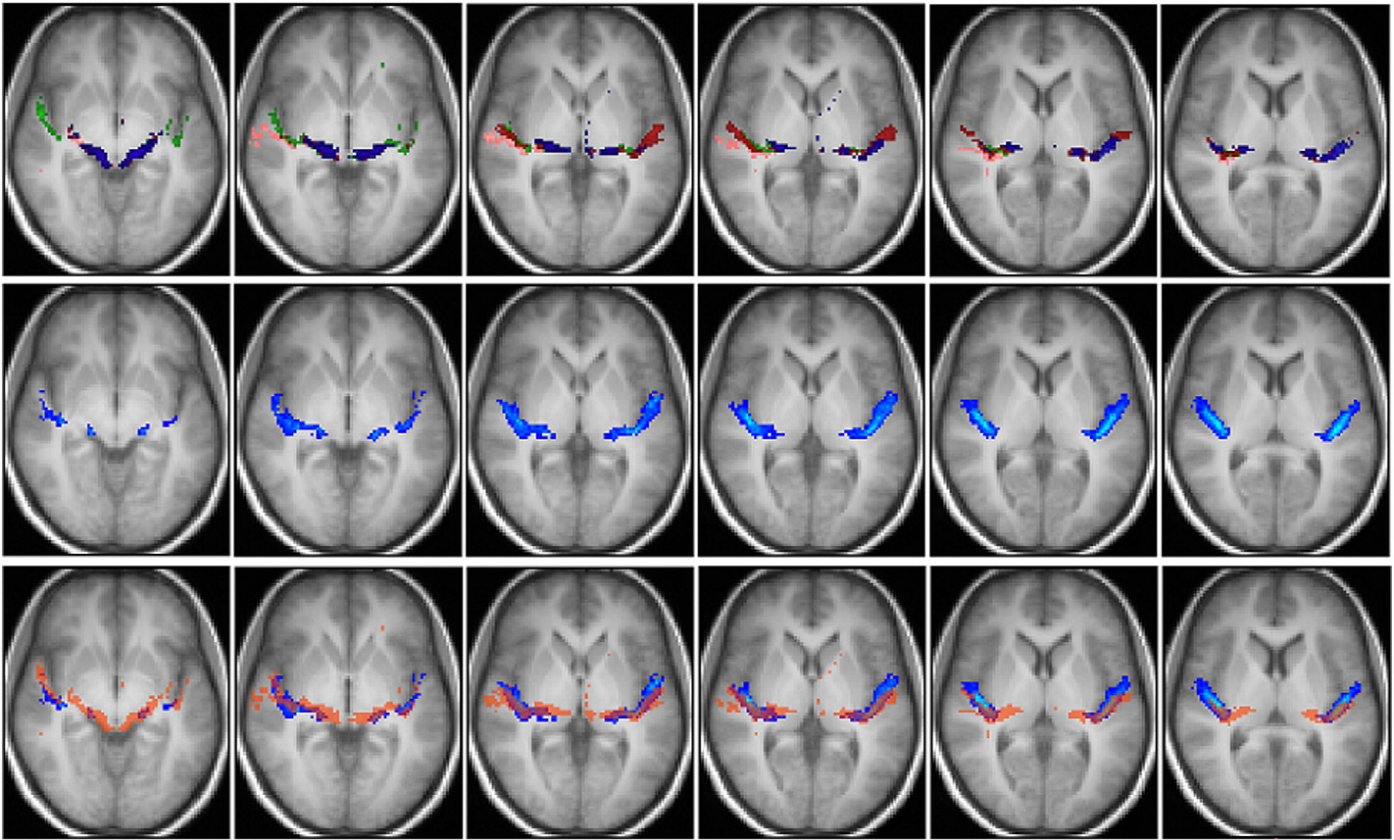
Top row: group fascicles connecting AC with IC via MGB overlaid onto the group-average T1 image in MNI space (radiological convention). Blue = *sound*–MGB–IC tracts, red = *pitch*–MGB–IC tracts, green = anterior *melody*–MGB–IC tracts, pink = posterior *melody*–MGB–IC tracts. Only tracts identified in more than 2 subjects are depicted. Middle row: tracts from a publicly available three-dimensional stereotaxic atlas based on data analysis of myelin-stained histological sections of ten postmortem brains (Burgel et al., 2006) overlaid onto the T1 group-average image of our subjects (radiological convention), depicted with a lower threshold of 14%. The slices selected are at the same superior–inferior coordinates as the slices of the top row. Bottom row: combination of the top and middle rows. Blue tracts are the atlas fascicles. The transparent red overlay represents the area occupied by all tractographic tracts (*sound*–MGB–IC, *pitch*–MGB–IC, anterior *melody*–MGB–IC, posterior *melody*–MGB–IC).

**Fig. 8:**
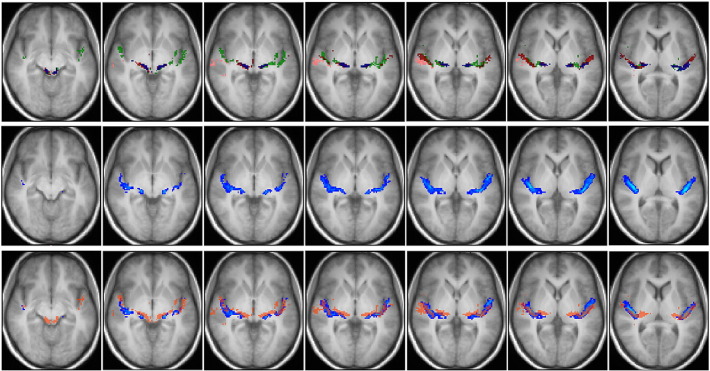
Top row: group fascicles connecting AC with IC directly overlaid onto the group-average T1 image in MNI space (radiological convention). Blue = *sound*–IC tracts, red = *pitch*–IC tracts, green = anterior *melody*–IC tracts, pink = posterior *melody*–IC tracts. Only tracts identified in more than 2 subjects are depicted. Middle row: tracts from a publicly available three-dimensional stereotaxic atlas based on data analysis of myelin-stained histological sections of ten postmortem brains (Burgel et al., 2006) overlaid onto the T1 group-average image of our subjects (radiological convention). Atlas tracts are depicted with a lower threshold of 14%. The slices selected are at the same superior–inferior coordinates as the slices of the top row. Bottom row: combination of the top and middle rows. Blue tracts are the atlas fascicles. The transparent red overlay represents the area occupied by all tractographic tracts (*sound*–IC, *pitch*–IC, anterior *melody*–IC, and posterior *melody*–IC).

**Fig. 9:**
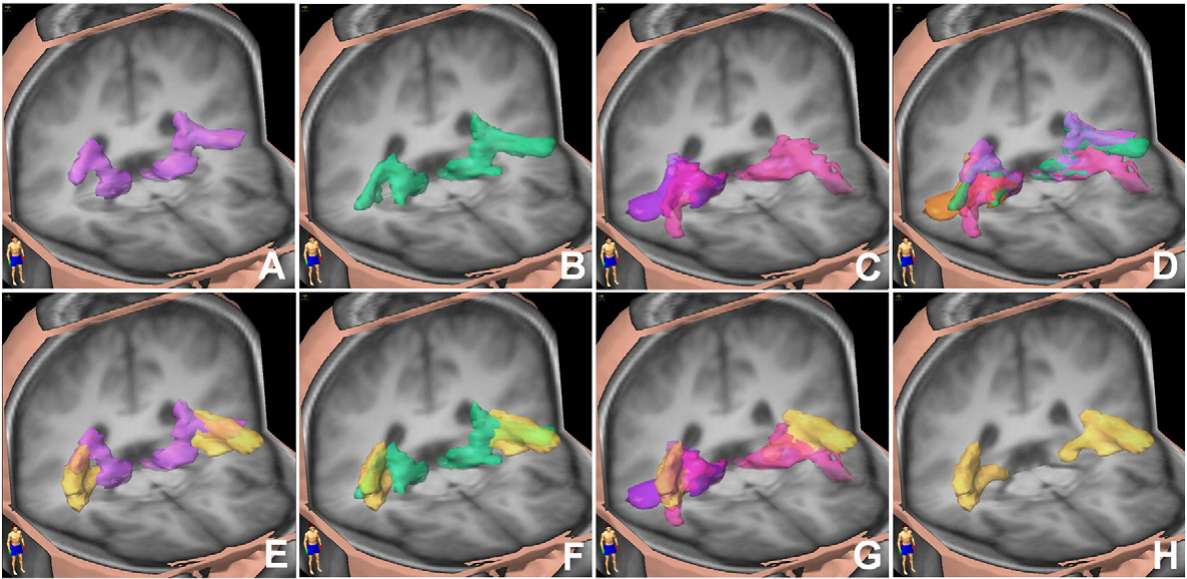
Three-dimensional images of the group T1 in MNI space, with overlays of the group auditory tracts connecting the auditory cortex with the IC via the MGB. The auditory tracts for each subject were thresholded at 0.05, and the group tracts were thresholded so that only tracts common to two or more subjects are displayed. A) light purple: tract connecting the *sound* area to IC via MGB; B) green: tract connecting the *pitch* area to IC via MGB; C) magenta: tract connecting anterior *melody* areas with IC via MGB, dark purple: tract connecting the right posterior *melody* area with IC via MGB; D) superposition of the tracts connecting *sound*, *pitch*, anterior *melody* and posterior *melody* with IC via MGB (colours are as in A, B, C, apart from posterior *melody* which is in orange); E), F), G) display the same tracts as in A), B), C), with the addition of (yellow) the tracts from the stereotactic histological atlas thresholded at 14%; H) yellow: tracts from the stereotactic histological atlas thresholded at 14%.

**Fig. 10:**
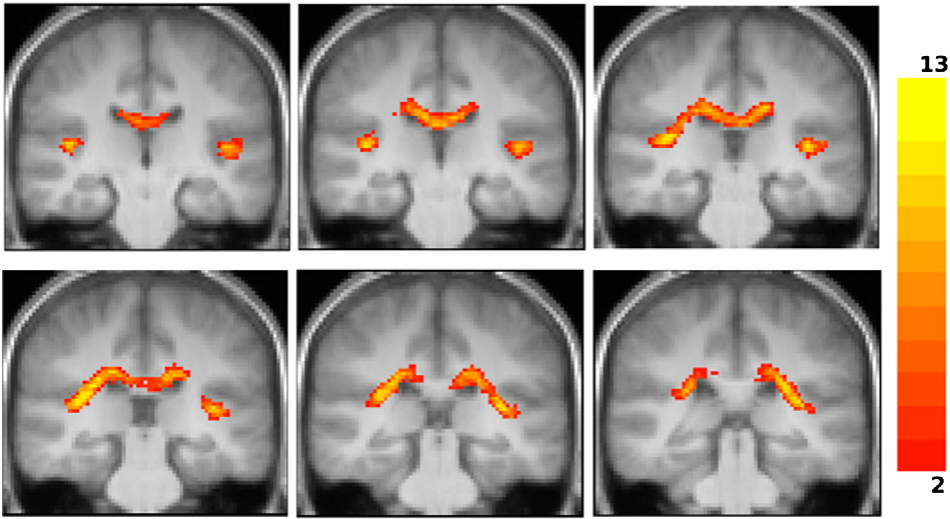
Group fascicles connecting right and left *sound* auditory cortexes via the corpus callosum overlaid onto the group-average T1 image of our subjects in MNI space (radiological convention). The signal intensity is proportional to the number of subjects in which the tracts were identified. Only tracts identified in two or more subjects are depicted.

#### Ascending versus descending projections

Fascicles connecting directly the AC with the IC have been reported as descending projections, while fascicles connecting the IC with the AC via the MGB have been reported to be ascending projections (Brodal, 1981). Group tracts connecting the three identified components of the AC directly to the IC nearly completely overlapped along most of their path with fibres connecting IC to AC via MGB. Some differences were visible at the level of the MGB, where IC–AC fascicles ran anterior to the MGB, while IC–MGB–AC fascicles included the IC–AC tracts and additional adjacent and more posterior voxels (Fig. 5).

#### Core and belt projections

Group fascicles for the *sound*, *pitch* and *melody* cortical areas overlapped with each other in the region between the IC and just before the *sound* area but then diverged into different tracts terminating at the *pitch* and *melody* areas, as shown in Fig. 6, in the top row of Fig. 7 and in Figs. 9(A, to D) for the fascicles passing through the MGB overlaid onto the group-average T1 image.

#### Tractography versus stereotaxic atlas

The identified group of fascicles connecting the IC with AC via MGB also matches the data from a publicly available three-dimensional stereotaxic atlas based on analysis of myelin-stained histological sections of ten postmortem brains (Burgel et al., 2006). This is shown in Fig. 7, where IC–MGB–AC group tracts obtained in this study (first row) and acoustic tracts from the atlas (middle row) are overlaid onto our group-average T1 image (bottom row), and in Fig. 9, where the atlas tracts are shown alone (Fig. 9H) and in combination with the various identified components of the auditory radiation (Figs. 9E, F, G). The match between the tractographic and atlas tracts is mostly evident in the bottom row of Fig. 7 where a red transparent area representing the region occupied by the AC–MGB–IC tractographic fascicles is overlaid onto the atlas fascicles (blue), and in Figs. 9E, F, and G. Similarly the tracts connecting *sound*, *pitch* and *melody* areas directly to IC match with the atlas fascicles (Fig. 8).

Group fascicles connecting right and left *sound* areas via the corpus callosum were also identified (Fig. 10), but no atlas data were available for comparison.

#### Tracts inter-subject variability

Tractography was successful in a maximum of 12 subjects for tracts via the MGB and for inter-hemispheric connections and of 9 subjects for tracts not including MGB (Table 2). The mean (standard deviation) of the group tracts was on average 2.0 (1.7) subjects out of 12 subjects (corresponding to a percentage of 17%) for the tracts connecting IC with AC via MGB and 1.6 (1.2) subjects out of 9 subjects (or 18%) for the tracts connecting AC directly to IC. For comparison, the mean (standard deviation) of the atlas tracts in the image space of the group T1 was 1.7 (1.7) subjects out of 10 subjects (corresponding to 17%). These measurements are evidence of a large inter-subject variability and of the agreement between tractographic and atlas data. The mean (standard deviation) of the group tracts for the fascicles connecting left and right *sound* areas was 3.4 (2.9) subjects out of 12 subjects (or 28%), suggesting a lower inter-subject variability for this specific fascicle, however no atlas data were available for this tract. The inter-subject variability is also evident in Fig. 6, Fig. 10 where the signal intensity is proportional to the number of subjects in which the tracts were identified.

## Discussion

Here we have localized subcortical auditory pathways using a combined tractographic-fMRI approach in a high proportion of healthy human subjects in vivo. The identified auditory pathway components connecting IC to portions of the AC specifically activated by *sound*, *pitch* and *melody* showed a close proximity with each other between IC and *sound* areas, with divergence between *sound* areas and higher order cortical areas. The fascicles showed a high degree of overlap with those obtained in human post-mortem data with cytoarchitectonic and myeloarchitectonic analyses. The small IC and MGB sizes and the limited MRI spatial resolution allowed only minor differences to be observed at the MGB level between projections connecting IC to AC via MGB and projections connecting AC directly to IC. No clear differentiation between ascending (via MGB) and descending (directly to IC) pathways can therefore be established by our data. Clear connections between right and left *sound* areas were also identified.

### Tracts identification

We hypothesised that the main limitation in the identification of the acoustic radiation was the crossing with major white matter fascicles (e.g. cortico-spinal tract, optic radiation), together with the complex intrinsic organization of the auditory pathways, the wide inter-subject variation in the location of the auditory cortex, and the mismatch between functional auditory cortices (and thus their functionally-determined subcortical linkages) and macro-anatomical landmarks (Baumann et al., 2013, Rademacher et al., 2001, Westbury et al., 1999). We therefore selected a low spatial resolution (2.3 × 2.3 × 2.3 mm^3^), and higher than standard diffusion-sensitising gradients (b-values 1400 s/mm^2^ instead of the more commonly used 1000 s/mm^2^), obtaining a small number of artefactual fascicles which were eliminated by simply thresholding the tracts with IndConn of 1% and 5%.

Even though our fMRI success rate was 100%, our methodology was not always successful in identifying the various components of the acoustic radiation. Our success rate in the IC-to-AC fibre identification was 71–75% at 1% IndConn threshold and 64–68% at 5% IndConn threshold. This reproducibility was higher than in the analogous tract identified by Crippa et al. (2010), who obtained a reproducibility of 50% using diffusion data with higher resolution (1.85 × 1.85 × 2mm^3^) and lower b-values (800 s/mm^2^).

Our success rate was even higher in the IC–MGB–AC fibres: 79–86% with IndConn threshold of either 1% or 5%.

The differences in success rate between tracts connecting IC directly to AC and fascicles passing through the MGB are most likely attributable to the difficulty of the methodology used in passing through grey matter areas, which is overcome by the selection of the MGB as waypoint mask.

Inter-hemispheric connections between left and right *sound* areas were also identified with a high success rate (86% of hemispheres).

### Comparison with 3D atlas

Baumann et al. (2013) showed that there is no easy correspondence between auditory core, belt and parabelt areas and anatomical landmarks in humans. Furthermore, Rademacher et al. (2001) showed that the location and volumes of auditory pathways and MGB vary considerably between individuals and hemispheres, and this spatial variability may lead to structural–functional mismatches in brain mapping studies based on the Talairach atlas. The location as well as inter-subject variability of our tractographic results matched well the location and inter-subject variability in this 3D tractographic atlas of Rademacher et al. (2001). This is not only reassuring but also somewhat surprising since we expected that the use of cortically-based seed regions (rather than adjacent white matter voxels or whole activation regions) might enhance the inter-subject consistency of our findings. Alternative seed procedures have also been associated with high intra- and inter-observer variability in tract localization (Cherubini et al., 2007, Guye et al., 2003), suggesting that wide inter-individual variation in fascicles might be matched with intrinsically wide variation in the location of cortical projection zones.

### Fascicles description

All tracts connecting IC with AC, either directly or via the MGB, follow a common path between the IC and the *sound* areas. Tracts to/from *pitch* and *melody* areas only diverge in the region between *sound* and *melody* cortices. This suggests an anatomical projection pattern similar to that described in tracer studies in other species, in which a core projection is surrounded by a belt/parabelt projection (Brodal, 1981, Lee and Winer, 2008, Rauschecker, 1998, Rouiller et al., 1991, Scott and Johnsrude, 2003).

The minor differences observed between tracts connecting IC to AC directly (supposedly descending projections) and IC to AC via MGB (supposedly ascending projections) most likely indicate that the image resolution of our data was inadequate to differentiate between ascending and descending pathways.

### Importance of present findings

In addition to localizing subcortical auditory pathways the present findings provide further information about the functional organization of the human auditory system. Direct linkages between subcortical nuclei and *pitch* and *melody* areas provide a substrate for ‘bypassing’ *sound* areas: this might support rapid processing of auditory information (e.g. auditory affective signals with high biological significance: Boatman and Kim, 2006). Alternatively, these parallel pathways might support cortico-subcortical circuitry involved in modulating and gating auditory information (Suga, 2008). Further studies are required to explore these possibilities. The present study has also demonstrated transcallosal linkages between left and right *sound* areas, consistent with findings in other species (Brodal, 1981), previous DTI (Westerhausen et al., 2009) and autoradiographic (Schmahmann and Pandya, 2006, Schmahmann et al., 2007) evidence and with the results of previous neuropsychological studies documenting specific auditory deficits following callosal interruption (Pollmann et al., 2002, Sugishita et al., 1995). Further work is needed to more fully assess the functional role of interhemispheric auditory projections particularly in the region of the splenium (Hofer and Frahm, 2006, Kim et al., 2008, Pollmann et al., 2002, Schmahmann and Pandya, 2006, Zarei et al., 2006).

### Clinical implications

Potential applications include the noninvasive assessment of tract morphology in disease states. For example, the prognosis for useful recovery of hearing following cochlear implantation is importantly influenced by the integrity of subcortical pathways (Vlastarakos et al., 2010). Severe forms of chronic tinnitus are likely to be perpetuated by central reorganization of cortical and subcortical pathways (Møller, 2003): a more detailed understanding of the anatomy of such changes could help guiding pathophysiological treatment strategies like repetitive transcranial stimulation (Langguth et al., 2010). Further applications could extend to neurosurgical planning: projections from the auditory periphery are bilateral (and therefore unilateral damage will not, in general, lead to deafness), however, complex cognitive processing such as that entailed in the analysis of human vocal signals is likely to require bilateral representation of auditory information. When planning neurosurgical procedures (e.g., for lesion resection), assessment of functionally significant white matter tracts is presently challenging; intra-operative electrical stimulation can demonstrate tract integrity but cannot delineate tract anatomy. The application of noninvasive anatomical modalities could potentially assist tract localization and thereby minimize post-operative deficits. Indeed, integration of DTI with standard intraoperative neuronavigation systems has been shown to be practical (Nimsky et al., 2006). To date the main clinical application of fMRI has been in localizing eloquent cortical areas (e.g. Hirsch et al., 2000, Majos et al., 2005, Roux et al., 1999). The present study suggests that fMRI may also aid DTI in localizing subcortical pathways, as already shown for the cortico-spinal tract (e.g. Cherubini et al., 2007). Our primary aim was to assess the robustness of the combination of fMRI and tractography for the identification of auditory pathways. Different fMRI paradigms could be selected to assess various components of the auditory system, including tonotopic and language-related fMRI paradigms. The anatomical convergence of auditory pathways demonstrated in this study suggests that an abbreviated auditory fMRI paradigm could be developed for clinical applications (e.g., Kovacs et al., 2006). This combined approach might be particularly useful where local anatomy has been distorted in the vicinity of a lesion (Tharin and Golby, 2007). Furthermore, the complex relation previously reported between sound and speech perception indicates that speech perception fMRI paradigms could also be used to identify high order auditory cortical areas (Scott and Johnsrude, 2003). The combined approach of fMRI and DTI might also be helpful in assessing more complex hypothesised projections between high order auditory areas and frontal, temporal and parietal areas (Scott and Johnsrude, 2003).

### Limitations

Volunteers were not specifically assessed for psychiatric disorders (although they were asked to confirm that they were healthy) or anatomical expertise (e.g. the expertise of professional musicians). Such conditions could affect the fMRI activation (Herholz and Zatorre, 2012). However, the exploration of single subject fMRI results showed that all subjects had patterns of activation similar to the activations in the group as a whole, suggesting that volunteers probably did not have psychiatric disorders and had comparable anatomical expertise.

fMRI activation of distinct auditory cortices is less robust and more variable for individual subjects than at group level: an important factor in any potential clinical applications.

Fine-grained separation of auditory pathways (e.g. ascending versus descending projections) based on DTI cannot be achieved at current image resolutions. At 3 T, higher resolution DTI (with high b-value) would currently require very long acquisition times (approximately 70–80 min) that are not tolerable for patients. The advent of improved gradient system, receiving coils, and scan acquisitions as those used in the human connectome project (www.humanconnectomeproject.org) and of ultra-high field (7 T) magnets will allow in the future acquisition of higher resolution diffusion MRI in reasonable times (Heidemann et al., 2010, www.humanconnectomeproject.org), but these systems are currently unavailable in clinical practice.

Fascicles connecting AC and IC directly were here visualised in fewer hemispheres than fascicles traversing MGB: while this may reflect intrinsic fibre distributions, it might also be at least partly attributable to the proximity of grey matter structures and crossing white matter fascicles.

From a practical perspective, both fMRI and DTI are labour-intensive modalities requiring extensive post hoc offline processing.

Finally, the present fMRI stimuli were pitch patterns: it is likely that other kinds of auditory signals are processed via distinct cortical mechanisms that may have different profiles of subcortical connectivity (e.g. tonotopy and the specialised signals of human speech). A similar caveat applies to the study of healthy subjects versus patients with neuro-otological disease.

Future work should build on the data presented here by investigating the processing of a range of auditory signals in clinical as well as healthy populations, and by combining customised fMRI protocols with high-resolution diffusion techniques (e.g. 7 Tesla-DTI, or techniques used in the human connectome project www.humanconnectomeproject.org) and dynamic connectivity-based techniques (e.g. dynamic causal modelling).
